# Mapping life’s disparity and evolutionary constraints in a geometric complexity space

**DOI:** 10.1126/sciadv.aea6945

**Published:** 2026-01-07

**Authors:** Guillaume Dera, Elise Nardin, Laurent Risser, Marius Albino, Quentin Garnier, Marion Kardacz, Léa Monge-Waleryszak

**Affiliations:** ^1^ Géosciences Environnement Toulouse (GET), CNRS UMR 5563, Université de Toulouse, IRD, CNES, Toulouse, France.; ^2^ Institut de Mathématiques de Toulouse (IMT), CNRS UMR 5219, Université de Toulouse, Toulouse, France.; ^3^ Centre de recherche sur la biodiversité et l’Environnement (CRBE), CNRS UMR 5300, Université de Toulouse, IRD, INP, Toulouse, France.; ^4^ Laboratoire des interactions plantes-microbes-environnement (LIPME), UMR CNRS-INRAE 2594/441, Université de Toulouse, Castanet-Tolosan, France.

## Abstract

The Earth’s biosphere exhibits a notable diversity of forms, yet the full morphological extent and limits of life remain largely unexplored. Here, we develop a geometric complexity space for comparing all known unicellular and multicellular phyla using fractal descriptors of the density and heterogeneity of body mass and structure. By applying this approach to a large set of extant biological shapes, we show that life exploits a tiny portion of structural possibilities, clustering around linear, rounded, and densely structured forms, while consistently avoiding complex heteromorphic ones. We show that this restriction results from deep physical, metabolic, and developmental limitations, shaped over geological time by the evolution of body size and ecological lifestyle. Our findings provide a global, quantitative perspective on the long-standing interplay between chance and necessity in evolution, with implications for the expected forms of life beyond Earth.

## Teaser

Life’s diversity is limited to a small set of shapes due to fundamental physical and evolutionary constraints.

## INTRODUCTION

From bacteria to insects or giant sequoias, living forms display a fascinating variability of geometries, body sizes, and complexity levels shaped throughout evolution (*1*–*4*). While some designs appear more common in nature due to evolutionary convergences across independent lineages (*5*–*8*), the overall morphological landscape of life on Earth still remains unexplored. Investigating the morphological limits of life and its global disparity patterns is yet crucial, not only for understanding the processes and constraints that govern the evolution of biodiversity on Earth but also for detecting possible life forms elsewhere in the universe (*5*, *9*).

Beyond phylogenetic, developmental, and environmental factors, whether contingent or necessary, that have shaped the variability and complexity of organismal forms through time (*10*), the question of universal morphological limits to life is rarely addressed or mainly through physicochemical constraints (*6*) and logical considerations (*11*). For example, forces such as surface tension constrain the smallest unicellular organisms to adopt spherical shapes, while gravity further influences the size, structure, and proportions of macroscopic terrestrial taxa (*6*, *12*, *13*). Intrinsically, living forms also rely on finely tuned surface-to-volume ratios to ensure thermodynamic trade-offs between external energy exchange and efficient internal resource transfer, storage, and assimilation, which are essential for metabolism (*14*, *15*). Combining geometric and metabolic models, Banavar *et al.* (*16*) thus predicted that multicellular organisms should range from arborescent forms with heterogeneous mass density and ramified structures to denser, homogeneous creatures such as animals. In other words, life would exploit a vast but bounded range of viable architectures, optimizing in different ways the flux and use of energy, matter, and information from the surrounding environment (*17*). However, the inexorability of these privileged morphological solutions is not fixed as life continually adapts to physical constraints through emerging ecological opportunities and morphological innovations (*18*).

**Fig. 1.**
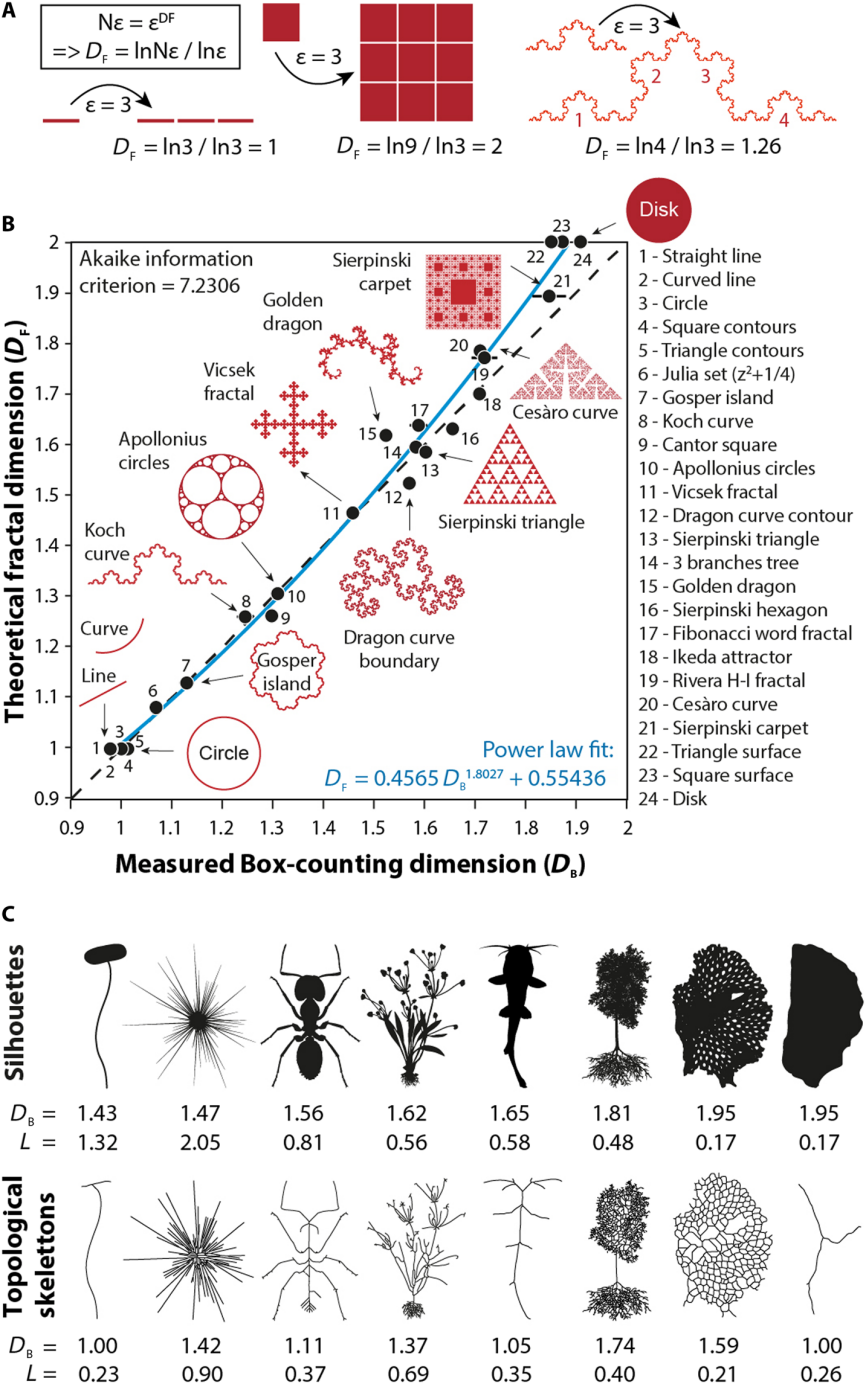
Concepts of fractal dimensions and topological skeleton of shapes. (**A**) Calculation of theoretical fractal dimension (*D*_*F*_) for self-similar geometrical objects. In this example, an arbitrary homothety (magnification by a factor ε = 3) is applied to a line, a square, and a Koch curve to quantify the number of resulting patterns *N*ε. (**B**) Comparison between the theoretical fractal dimensions (*D*_*F*_) of self-similar shapes and their empirical estimates (*D*_*B*_) obtained from box-counting analysis (see Materials and Methods). Because *D*_*B*_ values for denser shapes show slight deviations, we systematically corrected the values presented in this study using the indicated power-law adjustment. (**C**) Silhouettes and topological skeletons of organisms with their corresponding fractal dimension (*D*_*B*_) and lacunarity (*L*) values. Silhouettes are ranked left to right by increasing fractal dimension (*D*_*B*_). Fractal analysis of topological structures distinguishes cases with similar *D*_*B*_ values.

Here, we explore the open-ended nature of morphological evolution by comparing the possible and extant shapes of life, from prokaryotes to multicellular eukaryotes, regardless of body size, hierarchical complexity, and ecology. This global issue requires a comprehensive framework for morphological comparison across diverse phyla. Thanks to morphometrics and computational tools (*19*–*23*), this is traditionally addressed at lower taxonomic levels by creating morphospaces in which taxa are compared with other siblings or theoretically possible forms (*21*, *24*–*27*). Despite progress for specific clades (*28*–*31*), mapping the morphological spectrum of bacteria, metazoans, plants, and others into a single universal space remains challenging, particularly because of the use of idiographic phenotypic traits that are not operational to describe and compare taxa at the highest taxonomic ranks (*32*). To overcome these methodological impediments, we examine two fundamental characteristics of living forms: their internal topological structure and the surrounding body mass, whose external envelope separates the organism from its environment. First, we consider that any shape, biological or otherwise, can be described by a topological skeleton (or medial axis), a geometric abstraction composed of interlocking segments that captures the internal organization, continuity, and connectivity of subparts (Fig. 1C) (*33*, *34*). This minimalist thread-like structure, considered as the “mathematical backbone of shape,” provides information on the position and subdivision of developmental axes, symmetry, and overall branching patterns. In essence, it reflects the general structure that defines the global and invariant perceptual entity of shape known as “gestalt” (*35*, *36*). The biological grounding of the topological skeleton is not always straightforward, but in many cases (as with bilaterian organisms), the identified segments correspond to distinct spatial and functional units such as trunks, limbs, or appendages (*37*, *38*), at different nesting degrees (Fig. 1C). To some extent, these structural elements also present an internal integration and external isolation, typical of biological parts (*39*). From this mathematical abstraction, a wide range of silhouettes can be realized by locally varying the volume of body mass distributed along the topological skeleton. In this way, the geometry of the body envelope can be understood as the outcome of an invariant topological template undergoing variable transformations of its surrounding body mass (*35*). Although related, the two features provide distinct and complementary information about shape, since silhouettes with similar spatial properties (e.g., body mass density) can still be distinguished by their underlying topological skeletons (Fig. 1C).

Despite their global relevance, the topological skeleton and body mass of organisms cannot be readily quantified and compared using conventional morphometric approaches. Standard methods such as qualitative descriptions, linear measurements, or outlines and landmarks analyses require prior identifications and selections of anatomically homologous traits (*40*). However, this prerequisite does not apply here, given the numerical, developmental, and compositional variability of structural units under consideration. Furthermore, quantifying one-dimensional (1D), 2D, or 3D objects within the same mathematical framework remains challenging, particularly when the structural objects of interest demand specific topological descriptors (*41*). To overcome these limitations, we adopted an alternative approach based on the fractal properties of shape. Unlike most reductionist morphometric methods treating phenotype as a collection of selected traits (*42*), fractal descriptors provide a holistic perspective on the structure, geometry, and complexity of shape (*43*), by capturing its space-filling properties across scales, independently of the biological nature and identification of morphological traits. For self-similar shapes, these metrics follow a scaling rule formulated by a power law (*44*), *N*ε ∝ ε*^DF^* such that *D*_*F*_ = ln(*N*ε*)*/ln(ε), where *D*_*F*_ is the fractal dimension of shape and *N*ε is the number of replicated parts (or a measure) of that shape at an observation scale ε (or after a homothetic transformation ε) (Fig. 1A). This gives fractal dimensions *D*_*F*_ of 0 for a point, 1 for a line, 2 for a square, and 3 for a cube, while *D*_*F*_ varies between 1 and 2 for intermediates between lines and surfaces (Fig. 1B) (*45*). For irregular biological shapes, an alternative fractal dimension metric (*D*_*B*_) can be estimated using box-counting methods and complemented by a lacunarity parameter *L* expressing the overall shape heterogeneity (*43*, *44*, *46*–*49*). Together, *D*_*B*_ and *L* describe the spatial properties of body mass and structures in terms of density and heterogeneity, thereby facilitating the quantification and differentiation of complex biological shapes (Fig. 1C).

**Fig. 2.**
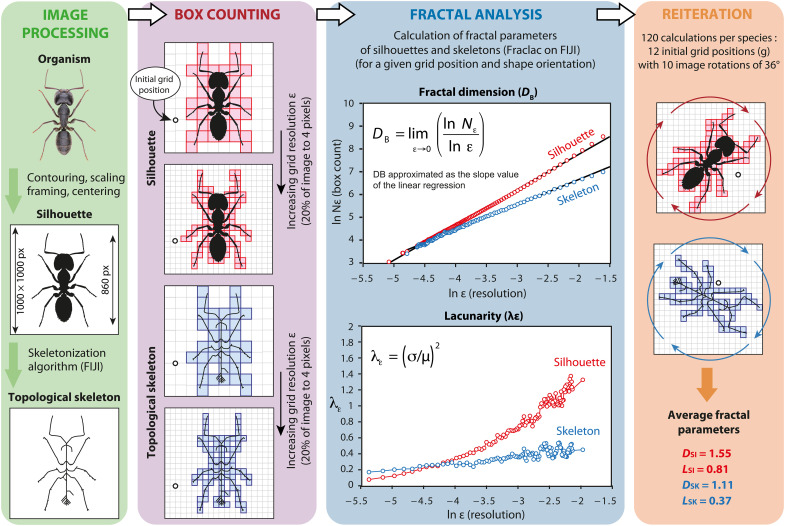
Protocol for measuring the fractal properties of the body mass and topological skeleton of organisms. Each species’ image is converted into a black silhouette and then reduced to a topological skeleton. Box-counting analyses are performed on both the silhouette and the skeleton by overlaying grids of progressively increasing resolution (ε). For each grid resolution and starting position, the number of boxes covering the shape (*N*ε) is recorded, along with the mean (μ) and SD (σ) of pixels per box. These values are used to calculate the fractal dimension (*D*_*B*_) of silhouettes and skeletons, as well as the overall lacunarity (*L*), derived from the average of lacunarity values (λε) across resolutions. The procedure is repeated 120 times by rotating the images in 36° increments and varying the initial grid position 12 times. Final parameters are averaged and reported as *D*_*SI*_, *D*_*SK*_, *L*_*SI*_, and *L*_*SK*_.

**Fig. 3.**
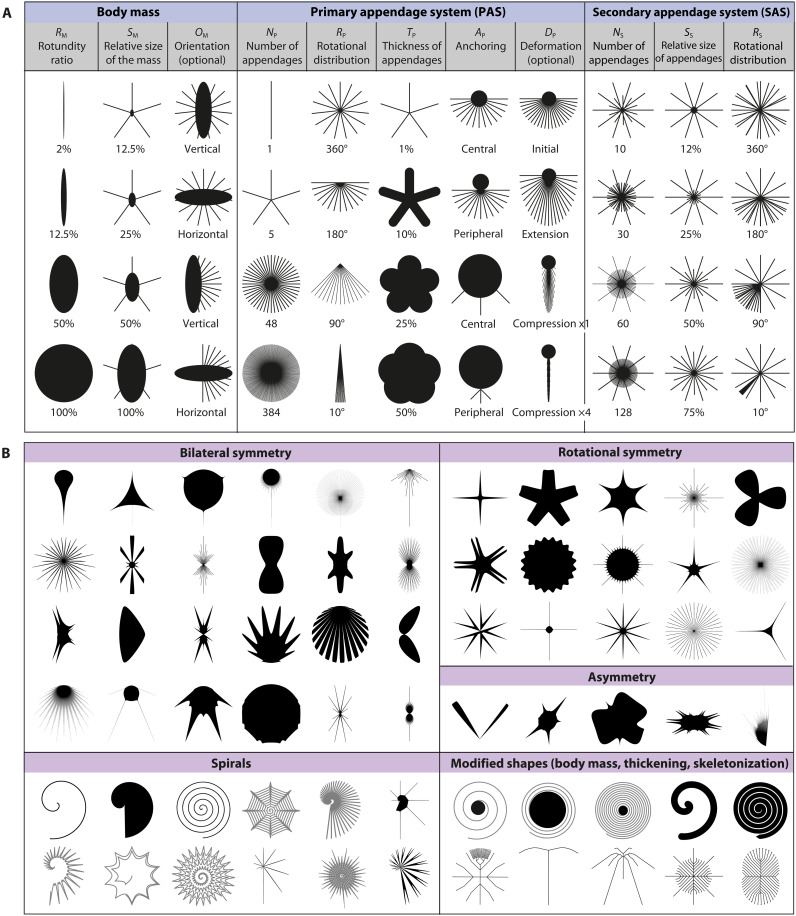
Examples of artificial biomorphs. (**A**) Shapes generated by vector drawing procedures on Adobe Illustrator. (**B**) Shapes generated using the Gielis’ superformula. These shape simulations are original works generated by the authors and are not subject to any external copyright holders.

To illustrate this structural and fractal approach to form, we analyzed 944 living specimens representing solitary, aggregated, and colonial forms of 839 extant species, including unicellular eubacteria, archaea, eucaryotes, and multicellular metazoans, plants, fungi, and algae (fig. S1 and data S1). Our sampling includes at least one, but more commonly 3 to 92 species per phylum reviewed in recent phylogenetic syntheses (*50*). This ensures a heuristic but representative overview of main body plans on Earth. Each image was converted into a binary, standardized, 2D silhouette summarizing the spatial distribution of body mass. Skeletonization algorithms were then applied to reveal the underlying structural patterns. For each specimen, we measured the *D*_*B*_ and *L* values of the silhouette and topological skeleton, resulting in four fractal parameters (*D*_*SI*_, *D*_*SK*_, *L*_*SI*_, and *L*_*SK*_) that quantify the spatial density and heterogeneity of body mass and structures (Fig. 2). To infer and explore heuristically the range of possible fractal combinations, we also an alyzed 15,389 artificial biomorphs generated using the Gielis’ superformula (*51*) and vector drawing methods (Fig. 3 and data S2).

## RESULTS

### Geometric complexity space

**Fig. 4.**
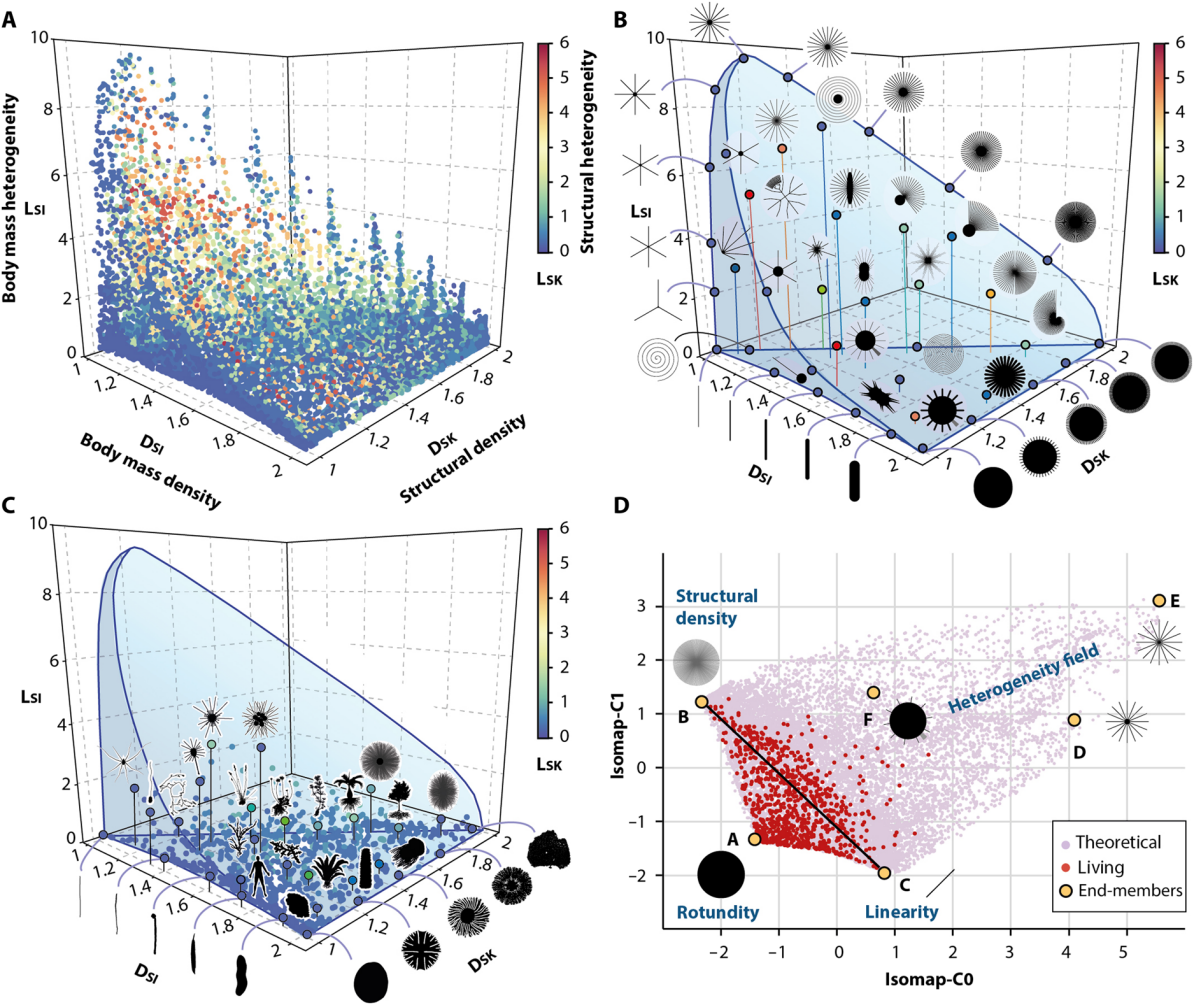
Geometric complexity space of modern organisms and artificial biomorphs. (**A**) Parameter space based on *D*_*SI*_, *D*_*SK*_, *L*_*SI*_, and *L*_*SK*_ values measured on 15,389 artificial and 944 living shapes. (**B**) Data points illustrating artificial shape variations along the axes. (**C**) Distribution of modern organisms with representative silhouettes. (**D**) Isomap ordination showing the restricted distribution of organisms within the field of possibilities. The black line separates isomorphic (bottom left) from heteromorphic shapes (top right).

When combined, the fractal parameters of artificial biomorphs and extant organisms result in a multivariate space that provides a comprehensive review of the variability and limits of living forms and their complexity (Fig. 4). Notably, this space synthesizes most spatial and scaling properties of biological forms, by implicitly capturing the number, proportion, position, and orientation of constitutive parts defining the overall shape conformation (*52*). In the absence of anatomical correspondences among the measured structures, and given the non-Euclidean and nongenerative (developmental) nature of fractal parameters, this space cannot yet be regarded as a “classical” theoretical morphospace (*22*, *25*, *53*, *54*); it is rather a space of “geometric complexity” (*55*). This distinction implies that organisms with different body plans may occupy nearby regions of the space due to similar space-filling properties (Fig. 1C) (*44*). At the taxonomical scale considered here, such conceptual trade-off favoring the statistical properties of spatial occupation is inevitable, but it does not compromise the broader morphological interpretation of the space, as in numerous complex systems, the number and spatial arrangement of local components remain tightly coupled with the overall pattern (*56*). Accordingly, the scale behaviors expressed by *D*_*SI*_, *D*_*SK*_, *L*_*SI*_, and *L*_*SK*_ may, to some extent, embody broader geometric principles underlying gestalt-level distinctions (e.g., continuity, proximity, similarity, closure, and symmetry) (*36*). Within this framework, *D*_*SI*_ quantifies body mass density, distinguishing mere lines such as cyanobacterial filaments (*D*_*SI*_ = 1) from dense, rounded organisms such as bacterial cells (*D*_*SI*_ = 2), with various elliptical intermediates in between. *D*_*SK*_ describes structural density and increases from 1 to 2, either by multiplying the number of appendages or by spreading and winding a single body structure over a large surface area. When *D*_*SI*_ ≈ *D*_*SK*_, the silhouettes equal their topological skeletons, so that the organisms resemble threadlike shapes (e.g., mycelium). Last, *L*_*SI*_ and *L_*SK*_* measure the degree of local variations in body mass and structures, distinguishing uniform shapes with invariant spatial properties (*L*_*SI*_ and *L*_*SK*_ = 0) from heteromorphic shapes with local differences (*L*_*SI*_ and *L*_*SK*_ up to ~10 and ~5, respectively). These heteromorphic shapes are divided into three end-members, which are better observed after Isomap ordination (Fig. 4D): (D) shapes with a very local concentration of body mass globally surrounded by thin and very sparse appendages; (E) threadlike equivalents with local concentrations of structures; (F) dense shapes combining both globally sparse and locally dense structures. These chimerical configurations produce considerable scale contrasts between their global and local properties by imposing trade-offs between dense body masses, thin structures, and voids.

Despite their simplicity and regular organization, heteromorphic biomorphs can be regarded as complex in a broad definition of complexity referring to the intrinsic diversity and variability of shape constituents (*57*–*59*). Within the information theory framework, this notion more specifically relates to the amount of information required to describe a system. In many biological systems (*55*), complexity is linked to the Shannon entropy *H*, which quantifies the average uncertainty associated with a variable (e.g., anatomical trait) that can take different values (or states) with given probability distributions (*60*). Ranging from 0 for perfectly regular patterns to 1 for random ones, entropy *H* is therefore interpreted as an index of disorder, which often correlates positively with fractal dimension (*D*_*B*_), but not necessarily with lacunarity (*L*) (*61*). Consequently, dense dendritic structures with numerous details and irregularities (e.g., bacterial mats with high *D*_*SI*_ and *D*_*SK*_ values) should be considered more complex than heteromorphic shapes. However, between regularity and chaos, life displays intermediate morphologies whose organized complexity may be more accurately described by a concave function that reaches its maximum at the balance point between order and disorder (*62*). More formally, this interpretation aligns more closely with the concept of LMC (López-Ruiz, Mancini, Calbet) complexity, which is grounded in the probabilistic description of physical systems (*63*). This definition posits that complexity *C* arises from the interplay between entropy *H* and disequilibrium *D* (i.e., *C = H* × *D*), in other words, between information contained in the system and its distance from the equiprobable distribution of the accessible states. Hence, we consider that the heteromorphic shapes have higher levels of LMC complexity.

Fundamentally, the geometric complexity space defined by the fractal parameters does not differ substantially from the “configurational complexity” space recently developed by Rock and Wills (*55*). Configurational complexity captures the organization of constituents, typically serial homologues, within a biological system (e.g., trilobites’ thoracic segments and mammals’ vertebrae) (*55*, *59*, *64*). It can be quantified by evaluating the variability and combinatorial logic of three independent descriptive axes (*55*, *58*, *65*): the number of parts, their differentiation, and the regularity of this differentiation. In the geometric complexity space, these principles are partly mirrored by the parameters *D*_*SK*_ (number of spatially distinct parts), *L*_*SI*_ (variation in the proportions of parts), and *L*_*SK*_ (irregularity in their spatial arrangement). Together with *D*_*SI*_, these fractal parameters provide thereby a comprehensive overview of the spatial conformation and complexity of shape, as well as the intrinsic geometric constraints it entails, as illustrated by the curved manifold (Fig. 4A) (*53*, *66*). However, configurational complexity approaches remain very complementary, being better suited to quantifying the complexity of homologous parts (*55*), that are often subsumed within the topological skeleton or overall body mass.

### A bounded space?

The gradual transition observed in the geometric complexity space between uniform and heteromorphic morphotypes is consistent with the metabolic model of Banavar *et al.* (*16*), which predicts intermediates between dense (animal-like) and heteromorphic (tree-like) organisms. Nevertheless, our results extend the spectrum of possible scaling/fractal properties (and overall resulting shape conformations) toward more linear variants (C) and three complex heteromorphic end-members (namely D, E, and F identified among biomorphic simulations; Fig. 4D). At a first glance, the seemingly continuous, finite, and curved nature of the manifold could reflect universal limits and constraints to the variability of geometric complexity (*17*). While such limits are mathematically valid for space domains where uniformity and scale invariance prevails (i.e., the triangular area between linear, rounded, densely structured morphotypes, such as 1 ≤ *D*_*SI*_, *D*_*SK*_ ≤ 2, and *L*_*SI*_ and *L*_*SK*_ = 0), this concept may be nuanced in terms of heterogeneity, since the range of lacunarity values is intrinsically linked to the shape resolution (*67*). This means that *L*_*SI*_ and *L*_*SK*_ could tend toward infinity as the scale contrast between the overall shape and its components increases (*68*). Furthermore, since the current space is heuristically derived from measured biomorphs, other shapes generated by alternative models with higher degrees of freedom could theoretically extend the spectrum of possible conformations (and their lacunarity) indefinitely. However, lacunarity is not expected to follow the same trend owing to its nonmonotonic behavior (*68*–*70*). Moreover, within a finite volume constrained by maximum resolution limits (as defined in the present study), the number of shape constituents is spatially restricted, and their interactions preclude certain positions and orientations, leading to forbidden configurations (*71*). Given that the upper bounds on *L*_*SI*_ and *L*_*SK*_ values could mainly result from the imposed range of scaling variations, we assume that the geometric complexity space likely underestimates the theoretical space of all possibilities. Nevertheless, it provides a meaningful and physically tangible approximation of the underlying structure, suitable for analyzing geometric complexity patterns across both microscopic and macroscopic scales.

As the specimens are reduced to 2D silhouettes, we acknowledge that the measured parameters fail to capture the true fractal (volumetric) properties of organisms. This loss of morphological information along the third orthogonal axis remains a recurrent issue in many morphometric studies, regardless of the analytic framework used (*72*, *73*). Overcoming this limitation would require major methodological advances combining 3D scanning of a wide diversity of organisms (*74*), modeling of 3D topological skeletons (*33*, *38*), and 3D fractal analyses (*75*). Pending “an escape from *Flatland*” (*73*), we can only anticipate that integrating such data will extend the range of *D*_*SI*_ and *D*_*SK*_ values from 1 to 3, thereby improving our ability to discriminate between planar and volumetric forms (e.g., a flat bacterial mat from a spherical prokaryotic cell). *L*_*SI*_ and *L*_*SK*_ are also likely to be affected, particularly in heterogeneous shapes such as trees, which combine linear (branches), planar (leaves), and volumetric (trunk) components (whose superposition tends to produce denser 2D projections). Despite this limitation, we argue that the current space offers a suitable framework for measuring and studying first-order complexity patterns, given the strong correlations measured between 2D and 3D fractal dimensions (*76*).

### Distribution and limits of living forms

**Fig. 5.**
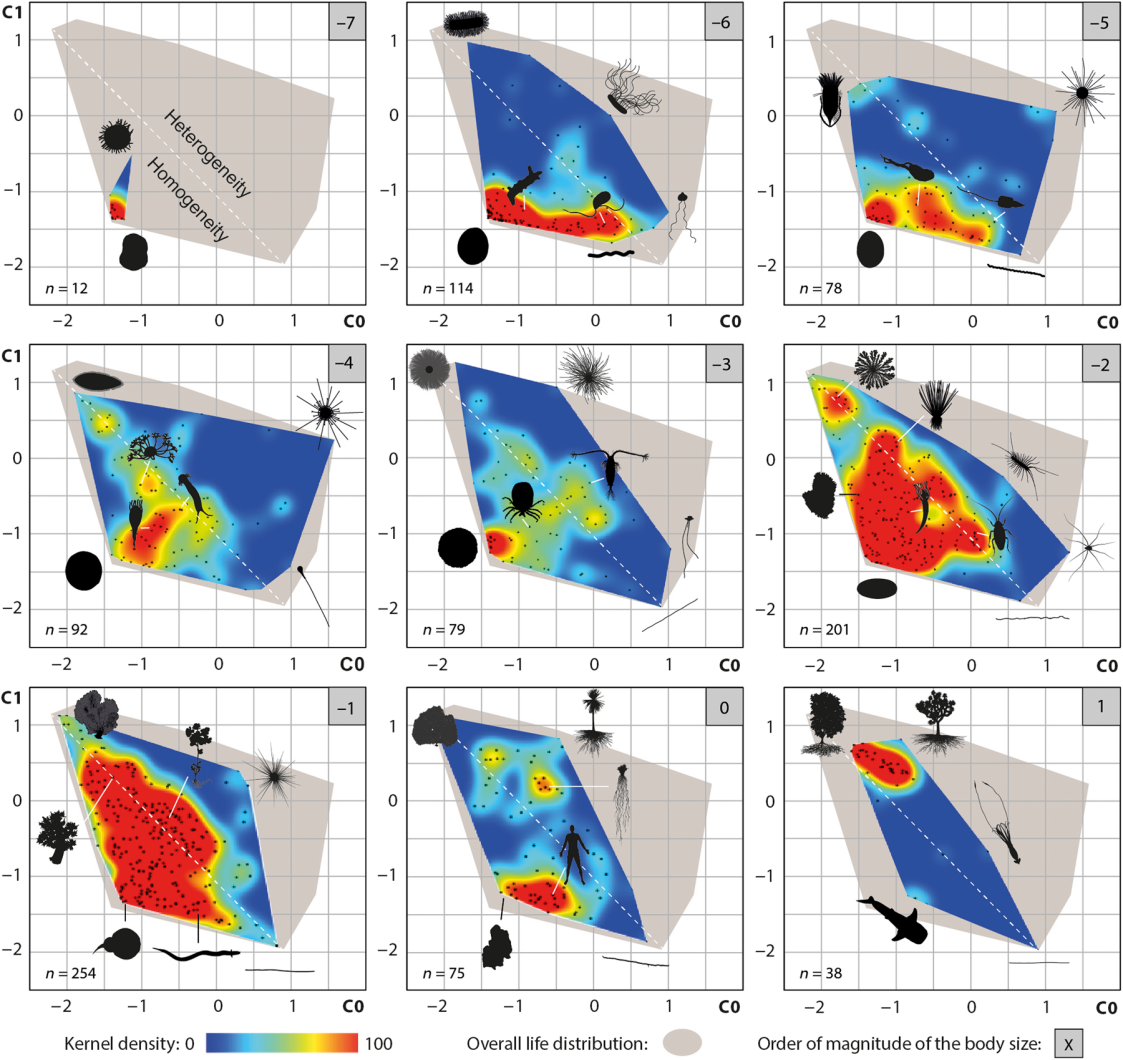
Distribution of organisms in geometric complexity space across body sizes. Body size is expressed in order of magnitude (log meters). Colored areas represent kernel density maps of the data points, while gray areas indicate the full range of living forms in the Isomap ordination (see Fig. 4D). The white dashed line approximately separates isomorphic (bottom left) from heteromorphic shapes (top right).

Using bootstrapped ratios of generalized variances, we find that the extant organisms occupy only 0.4‰ (σ = 0.4) of the field of possibilities defined by the artificial biomorphs (table S1) (as discussed above, this value, even low, likely overestimates biological reality). Though life exploits almost all possible geometric complexity schemes between linear, rounded, and densely structured morphotypes, this low disparity is mainly due to the absence of highly heteromorphic, complex forms (Fig. 4, C and D). The most heteromorphic organisms in terms of mass (*L*_*SI*_ > 2) are unicellular eukaryotes (rhizarians and centrohelids), followed by certain arthropods or sea urchins (Figs. 4C and 5), all characterized by the presence of a small body mass supported by a mineralized envelope and surrounded by a few or many long, thin, radial appendages, spines, or pseudopods. Conversely, plants with dense roots and sparse aerial systems show the highest levels of structural heterogeneity (*L*_*SK*_ > 1). Obviously, our sampling is limited compared to the millions of species on Earth. Adding further taxa would perhaps broaden the dispersal of clades in the geometric complexity space. However, the overall distribution is unlikely to change substantially, as all body plans have been considered regardless of body size, ecology, and hierarchical complexity. In addition, bootstraps and rarefaction procedures were used to minimize the sampling bias.

**Fig. 6.**
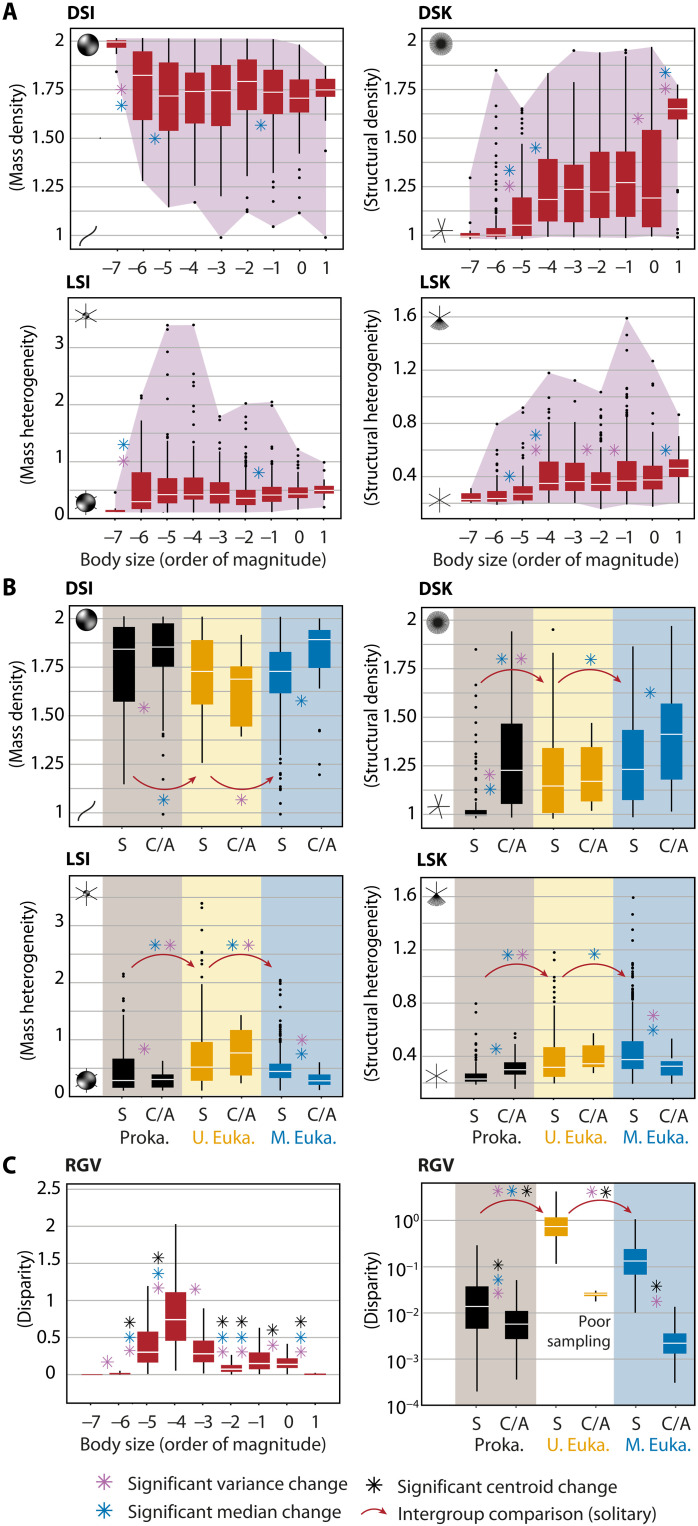
Variation in fractal parameters and disparity across body size and levels of hierarchical complexity. (**A**) Boxplots of *D*_*SI*_, *D*_*SK*_, *L*_*SI*_, and *L*_*SK*_ values across body size. Dots represent outliers and shaded purple areas indicate the full range of variation. (**B**) Boxplots of *D*_*SI*_, *D*_*SK*_, *L*_*SI*_, and *L*_*SK*_ across hierarchical complexity. Hierarchical complexity levels correspond to prokaryotes (Proka.), unicellular eukaryotes (U. Euka.), and multicellular eukaryotes (M. Euka.), each further divided into solitary (S) or colonial/aggregated (C/A) forms. (**C**) Boxplots of disparity estimates (excluding outliers), expressed as bootstrapped ratios of generalized variances (RGVs). Significant differences in variances, medians, and centroids (*P* < 0.05) are denoted by asterisks (see Table 1 and tables S1 and S2).

Supported by statistical tests (Table 1 and tables S1 and S2), our analysis reveals that the patterns of geometric complexity and resulting disparity estimates are strongly related to body size (Figs. 5 and 6A). At the smallest scale of life (10^−7^ m), prokaryotes are restricted to simple rounded cells, but from 10^−6^ m onward, some tend to elongate or to develop one or more flagella and filaments. At 10^−5^ m, the diversity of unicellular eukaryotes extends space occupation toward more linear and densely structured regions, while few heteromorphic shapes begin to appear. This trend continues up to a significant disparity peak at 10^−4^ m (Fig. 6C), associated with previously unrepresented protist phyla with strong mass and structural heterogeneities. At this scale, the median life shifts further toward forms of intermediate complexity characterized by dense body mass and multiple appendages. This occupation is maintained at 10^−3^ m, but as multicellular organisms and bacterial colonial forms gradually replace unicellular eukaryotes, the most heteromorphic shapes disappear, decreasing the disparity levels at 10^−2^ m. This decrease in disparity is reversed by a slight increase at 10^−1^ m, mainly due to terrestrial plants or animals with higher levels of structural heterogeneity. At metric and decametric scales, space occupation decreases, first with a separation of shapes into massive and ramified poles and then with the almost complete disappearance of massive shapes (except in aquatic environments). At this stage, most shapes are therefore arborescent. Although larger organisms have not been sampled, threadlike forms are expected to dominate at greater scales, as observed in kilometric fungal networks.

**Table 1.** Pairwise comparison of groups as a function of body size and hierarchical complexity. The significant differences (*P* < 0.05) are in bold. U., unicellular; M., multicellular; S, solitary; C/A, colonial or aggregated.

Groups	Sampling	Multivariate Shapiro-Wilk (*P* value)	Multivariate Wilcoxon test (*P* value)	PERMANOVA pairwise test (*P* value)	Box’s M test (*P* value)	ANOSIM test (*P* value)
**Body size (order of magnitude)**	*n*	H0: Normality	H0: Equal medians	H0: Equal centroids	H0: Equal covariance matrix	H0: Group similarity
−7	12	0.337				
			0.051	0.078	**<0.001**	0.991
−6	114	**<0.001**				
			**<0.001**	**0.001**	**<0.001**	**0.012**
−5	78	**<0.001**				
			**0.042**	**0.003**	**<0.001**	**0.009**
−4	92	**<0.001**				
			0.182	0.562	**<0.001**	0.919
−3	79	**<0.001**				
			**0.047**	**0.041**	**<0.001**	**0.033**
−2	201	**<0.001**				
			**0.034**	**0.001**	**<0.001**	**0.045**
−1	254	**<0.001**				
			0.991	**0.012**	**0.027**	0.173
0	75	**<0.001**				
			**<0.001**	**0.001**	**<0.001**	**<0.001**
1	38	**<0.001**				
**Hierarchical complexity level**	*n*	H0: Normality	H0: Equal medians	H0: Equal centroids	H0: Equal covariance matrix	H0: Similarity of groups
Prokaryotes (S)	116	**<0.001**				
			**<0.001**	**0.001**	**<0.001**	**<0.001**
Prokaryotes (C/A)	94	**<0.001**				
U. eukaryotes (S)	127	**<0.001**				
			1.000	0.131	0.845	0.472
U. eukaryotes (C/A)	5	**0.040**				
M. eukaryotes (S)	555	**<0.001**				
			1.000	**0.001**	**<0.001**	0.219
M. eukaryotes (C/A)	47	**0.001**				
Prokaryotes (S)	116	**<0.001**				
			**<0.001**	**0.001**	**<0.001**	**<0.001**
U. eukaryotes (S)	127	**<0.001**				
			1.000	**0.001**	**<0.001**	**<0.001**
M. eukaryotes (S)	555	**<0.001**				

**Fig. 7.**
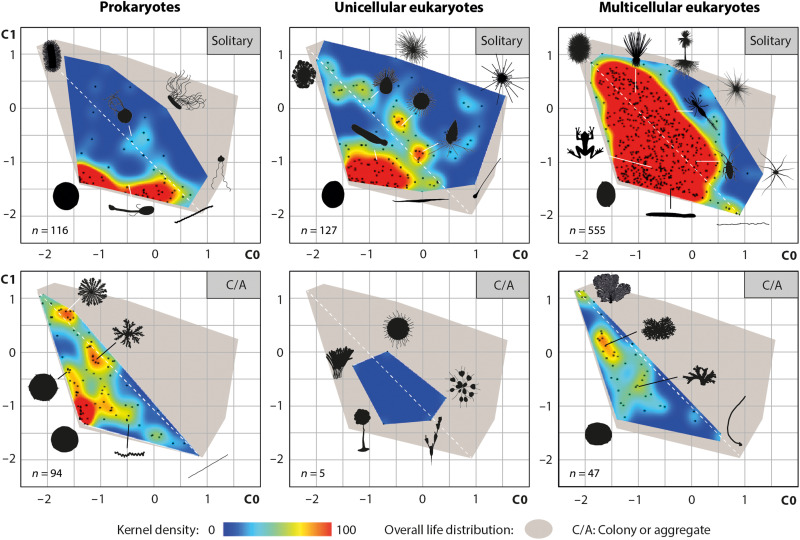
Distribution of organisms in geometric complexity space across hierarchical complexity. Legend as in Fig. 5.

Another point to highlight is that increasing hierarchical complexity does not systematically result in higher levels of disparity in geometric complexity terms. A notable rise is observed only at the transition from prokaryotes to unicellular eukaryotes (Figs. 6, B and C, and 7), as a probable consequence of more complex cytoskeletons and biomineralization processes favoring both the deformation and maintenance of body envelopes, as well as the deployment of filaments, pseudopods, or spines (*77*). Despite higher levels of structural density and heterogeneity, the evolution of eukaryotes toward multicellularity marks, however, a significant reduction in disparity (Figs. 6, B and C, and 7). As this pattern also applies to the transitions from solitary to colonial life in both bacteria and metazoans, we hypothesize that, whether at the scale of cells or individuals, this decrease reflects stronger morphogenetic constraints, particularly in terms of cohesion, cooperation, and self-organization (*11*).

## DISCUSSION

### What shapes living forms?

**Fig. 8.**
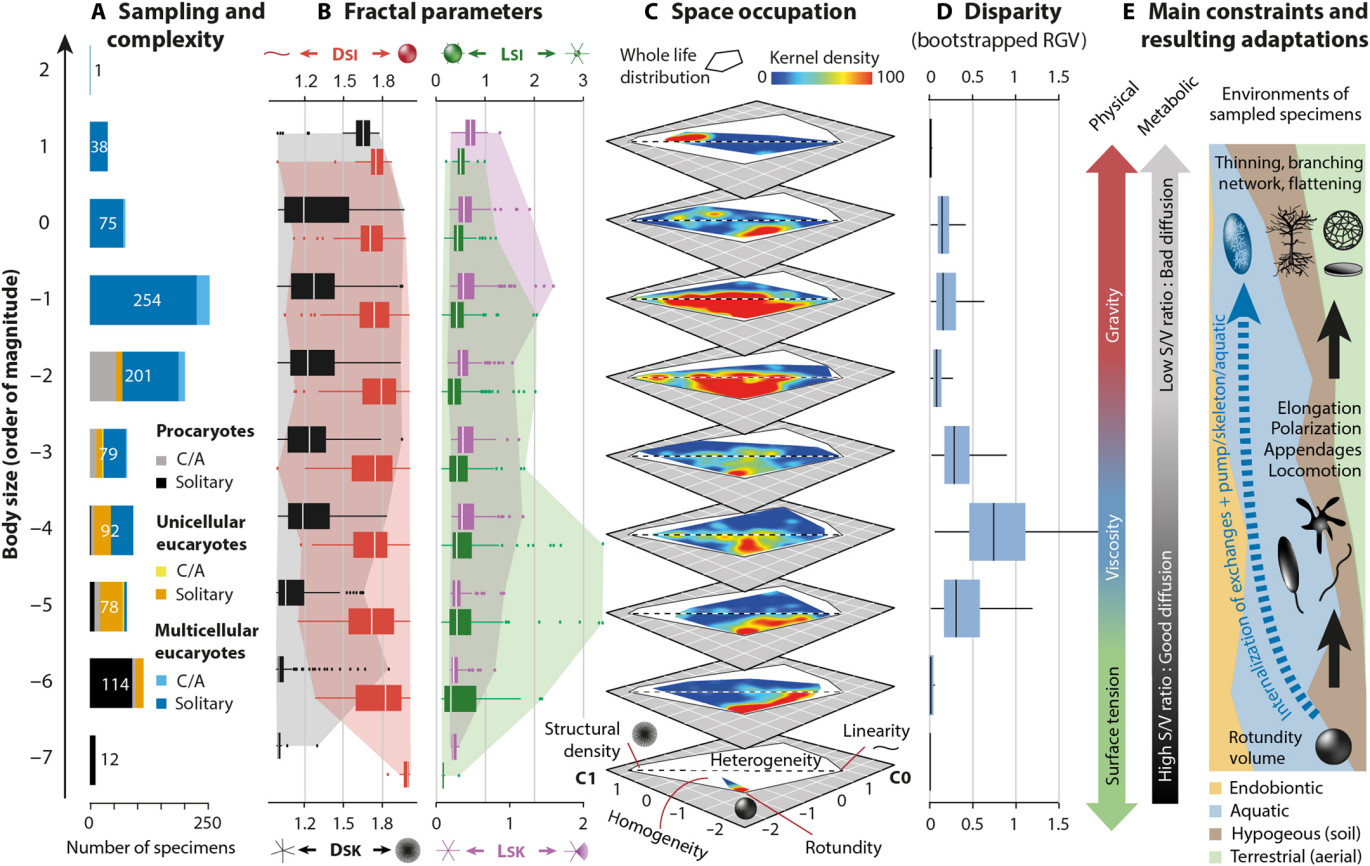
Geometric complexity and disparity of living forms across body size and constraints. (**A**) Sampling and hierarchical complexity of studied organisms (C/A, colonial/aggregated specimens). (**B**) Boxplots of fractal parameters with outliers (dots) and maximum variation ranges (colored areas). (**C**) Kernel density maps of living forms within the geometric complexity space (as in Fig. 5). The dashed line approximately separates uniform from heteromorphic shapes. (**D**) Disparity estimates calculated as RGVs with 1000 bootstraps and rarefactions to 50 samples. (**E**) Main physical and metabolic constraints acting on a growing shape (S/V, surface/volume ratio). The relative ecology of the studied specimens is indicated. Black shapes represent expected adaptations to theoretical constraints, while the dashed blue arrow indicates alternative innovations compensating for these modifications.

Our findings raise key questions about life evolution: Why do the geometric complexity and structural disparity of organisms vary so markedly with body size, and why are highly heteromorphic and complex forms so rare in nature? To address these questions, we develop an approach grounded in Seilacher’s principles of constructional morphology (*78*), which considers organismal forms as the outcomes of synergistic processes involving three morphodynamical factors: function, fabrication, and (genetic) computation, all modulated by environmental constraints. In the following, we examine the respective roles of these factors and show how their dependence on body size and environment imposes some directional trends and contributes to the exclusion of complex heteromorphic shapes (Fig. 8).

#### Function

Many authors define living forms as thermodynamic dissipative systems that combine essential properties such as compartmentalization, metabolism, homeostasis, reproduction, and learning (*79*, *80*). Once combined, these functions maintain the physical and informational (genetic) integrity of organisms, at both individual and phylogenetic scales. Hence, multiple morphological adaptations have been developed to harvest energy, matter, and information from the external environment, to transfer, store, and transduce these resources internally, before exporting waste, heat, genetic, chemical and physical information outside (*78*, *79*). In particular, the geometry of body envelopes has a profound effect on metabolic efficiency, as surface area modulates external exchange, while volume controls internal transfers (*14*, *81*). Since surface area increases proportionally less than volume for a given growing shape (by powers of 2 and 3, respectively), large organisms have to adapt their shapes to maintain a vital balance between external and internal energy flows (a trend also evident in allometric growth). In this context, spherical shapes are efficient at the microscale due to an optimal surface/volume (S/V) ratio, whereas elongation, flattening, or appendage multiplication become necessary to maintain a reliable S/V ratio at the macroscale (Fig. 8E) (*81*–*83*). Our results fully support this metabolic scaling law, as evidenced by the rising abundance of more linear and then densely structured organisms at larger scales (Fig. 8, B and C) (*83*). However, our results also demonstrate that the metabolic trend has been counteracted repeatedly throughout evolution, with dense elliptical shapes persisting at almost all body scales below 10 m (Fig. 8C). This conservation was certainly facilitated by key innovations that collectively maintained suitable energetic balances (*16*, *84*): (i) internal pumps (e.g., heart) and circulatory systems to enhance transfer, (ii) internalization and complexification of exchange surfaces (e.g., gut and gills), and (iii) nesting of energy fluxes by increasing hierarchical complexity. In a conceptual communication efficiency framework, such adaptations could also explain the maintenance of long linear shapes (e.g., parasitic worms) that generally suffer from limitations in resource diffusion (*85*).

Beyond their metabolic roles, limbs and appendages perform multiple functions essential for species survival. Their fitness reflects both ecological aspects related to the trophic position within food webs (e.g., locomotion, feeding, predation, defense, and sensory activity) and reproductive functions (e.g., fertilization, dispersal, and sexual attraction). However, unlike in certain morphospaces where form can be directly associated with a specific ecological function or with combinations of functions optimized according to a Pareto law (*54*, *86*–*88*), translating the geometric complexity space into a general adaptive landscape remains challenging. This difficulty arises because the functional role and efficiency of morphological attributes vary with environmental context, body size, and evolutionary history [e.g., exaptation (*89*)]. For instance, aquatic organisms with similar shapes exhibit different hydrodynamic properties (e.g., Reynolds number, drag coefficient, and velocity) depending on their body size (*90*). From a macroevolutionary perspective where species coevolve within increasingly complex food webs and changing environments (*91*), exploring the link between geometric complexity, ecology, and body size would be particularly insightful (*64*). However, such an analysis would require a more extensive and dedicated approach beyond the scope of the present study. At this stage, only preliminary observations can be advanced. For instance, most autotrophic organisms are typically either devoid of appendages at the smallest scales of life (e.g., prokaryotes) or characterized by multiple branching structures at larger scales (e.g., trees or multicellular algae). Despite their contrasting architectures, both configurations are consistent with a sessile lifestyle and a relative maximization of passive resource capture. In contrast, heterotrophic organisms such as metazoans (typically occupying intermediate body size ranges) require active foraging. To this end, cumbersome structures and complex exchange surfaces tend to be internalized within compact body envelopes (*92*), complemented by a smaller number of specialized organs for locomotion, detection, and feeding—features that correspond to low or moderate levels of heteromorphy. Likewise, the inexistence of complex heteromorphic organisms could reflect the absence of adaptive (functional) benefits, as elongated, slender, and sparse appendages become mechanically unviable when reduced below the minimum thickness required to house internal biological machinery (*3*).

#### Fabrication

Living matter is shaped by implacable physicochemical forces known as fabrication constraints (*78*). Throughout growth, they pose material and architectural challenges to cope with biomechanical stresses, balance physicochemical gradients, and face material availability imposed by the environment. Given the ecological variability of organisms, tackling this issue globally remains complex, but general trends can be identified as the influence of specific forces gradually changes with object dimension (Fig. 8E) (*93*). At microscales (<10^−4^ m), surface tension, Brownian motion or van der Waals forces dominate and shape the surfaces (*93*), making spherical envelops stable and linear or ramified shapes more unstable (*6*). Conversely, at macroscales, gravity becomes dominant and acts on volumes so that, in agreement with our results, rounded organisms several meters long became rare because they would collapse under their own weight (*13*). Apart from living in water where gravity is compensated for, overcoming this body size challenge on land requires drastic morphological adaptations, such as thinning, flattening, networking, or branching patterns seen in trees (*92*). This last solution divides the mass along increasingly fine structures so that the effect of gravity is reduced at each structural level. However, this adaptation requires more rigid or flexible biomaterials, whether organic or mineral. When arranged into endo- or exoskeletons, such biomaterials could also account for the persistence of dense forms at multimeter scales (e.g., giant mammals) and enable certain heteromorphic shapes at lower scales (e.g., rhizarians, arthropods, and echinoderms). However, limitations are expected because, whether flexible or rigid, ultrathin and elongated appendages are subject to biomechanical stresses, preventing the realization of more complex heteromorphic forms.

Defining a global scalar threshold for the relative influence of physical forces remains challenging, given the ecological specificities of taxa. However, our data reveal an intriguing pattern: The highest disparity levels of life and the emergence of the most heteromorphic taxa occur in isotropic aquatic environments around 10^−4^ m (Fig. 8D). This scale broadly corresponds to the transition between the micro- and macroscopic worlds, where surface tension gives way to viscosity and gravitational influences (*93*). This physical confusion has long been considered detrimental to the development of stable living shapes, justifying a presumed diversity gap at this scale (*92*). With the presence of cytoskeletons and mineralized tests, mass heterogeneity appears however to be an adaptation to this ambiguity (Fig. 8B). In contrast, the slight disparity peak observed at 10^−1^ m is more likely due to a prevalence of structural heterogeneity. At this scale (Fig. 8, D and E), gravity prevails and imposes anisotropic conditions (*93*), forcing organisms to rest on contact surfaces between solid, liquid, or gaseous media. In this context, developing structural heterogeneity becomes relevant, as the differentiation, polarization, and specializations of body parts enhance tropic responses to concurrent constraints (*92*). Ultimately, this suggests that heteromorphy emerges from physical dualities, whether in isotropic or anisotropic conditions.

#### Computation

**Fig. 9.**
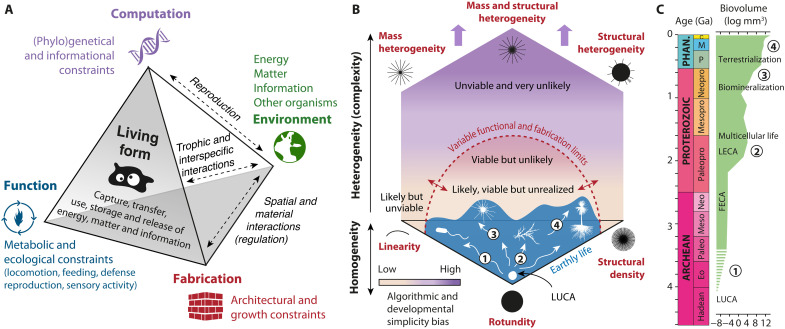
Influence of morphodynamical factors on form evolution over time. (**A**) Schematic representation of morphodynamical interactions influencing organismal form (*78*). (**B**) Schematic distribution of unlikely (purple), likely but unrealized (yellow), and extant living forms (blue) within a geometric complexity space of physically tangible possibilities (the upper arrows indicate theoretical extent toward incommensurable complexity). The limits imposed by function (metabolic) and fabrication (physical) constraints are inherently scale- and environment dependent, whereas the computation (genetic) constraints contribute to space anisotropy. The sequence of morphodynamical expansions linked to changes in body size and ecological lifestyle is indicated by numbers. (**C**) Timing of morphodynamical events (numbered as in B) relative to key evolutionary milestones and increases in body size (*4*) throughout geological time: (i) prokaryotic cell elongation; (ii) rise in structural density and heterogeneity due to eukaryotic cytoskeleton and multicellularity; (iii) first protists with high mass heterogeneity due to biomineralization; (iv) diversification of terrestrial organisms with pronounced structural heterogeneity (e.g., plants). LUCA, Last Universal Common Ancestor; FECA/LECA, First/Last Eukaryotic Common Ancestor; Eo, Eoarchean, Paleo, Paleoarchean; Meso, Mesoarchean; Neo, Neoarchean, Paleopro, Paleoproterozoic; Mesopro, Mesoproterozoic, Neopro, Neoproterozoic; P, Paleozoic; M, Mesozoic; C, Cenozoic.

Beyond previous constraints (Fig. 9A), organismal form is foremost the spatial and material expression of a set of generative instructions inherited from a long phylogenetic history shaped by gene mutation, heredity, genetic derive, and selection (*78*). From molecular assembly to overall conformation, the genotype encodes and regulates multiple aspects of organisms and their development by influencing protein sequences, cell behavior, and tissue biophysics (*94*). However, genome variations do not translate directly into phenotype variations, owing to the interplay of several factors operating at genetic, developmental, and environmental levels (e.g., epistasis, pleiotropy, genetic redundancy, neutral mutation, gene regulatory networks, developmental robustness, phenotypic plasticity, or epigenetic regulation) (*95*–*98*). The relationship between the space of genes and the space of phenotypic variations is therefore not linear, highly contextual, and intricately structured. This abstract link, known as the “genotype-phenotype map” (GPM) (*99*), lies at the heart of recent debates about evolutionary dynamics (*97*, *100*, *101*), as its complexity and robustness play key roles in determining the evolvability of organisms (*102*, *103*). Recently, this notion has gained considerable attention within the field of algorithmic information theory. For instance, recent theoretical models describing the GPMs of molecular structures have demonstrated that, in the absence of natural selection, random mutation processes would statistically favor simple phenotypes because, on average, simple outcomes require less information to encode, have simple and robust GPMs, and can arise through different developmental pathways (*104*). At larger morphological scales, this algorithmic simplicity bias could also account for the prevalence in nature of organisms with repetitive patterns based on symmetry, rotation, and fractality (*103*). To the opposite, complex phenotypes demand highly specific developmental processes that act locally and rely on multiple physical, chemical, and cellular mechanisms, all requiring highly accurate information signaling (*11*, *105*). This requirement implies finely tuned and intricate GPMs that are particularly sensitive to noise (*94*, *102*, *103*). As a result, random mutations during evolution would tend to reduce the complexity of already complex phenotypes, a trend recently demonstrated both theoretically using Dawkins’s biomorphs (*106*) and empirically through models of leaf and tooth development (*107*, *108*). In turn, greater phenotype complexity would confer a higher potential for generating substantial innovations (*102*). As observed in recent bird data (*109*), a corollary of this principle is that simple phenotypes are expected to be more abundant and to evolve more gradually within a given morphological range, whereas complex forms should be rarer yet capable of producing discontinuous, patchy occupations of morphospace (*110*). Of course, transforming the linear carrier of genetic information (e.g., DNA) into multidimensional architectures could, in theory, mitigate these limitations by expanding the genotypic space and the complexity of GPMs (*11*). However, challenges in evolvability, computation, and thermodynamics would persist. Even if selective pressure cannot be discounted and probably operated concurrently under both functional and fabricational constraints, the simplicity bias might offer a compelling explanation for the large-scale absence of highly complex shapes in the heteromorphic domains of the geometric complexity space.

Invoking a general bias toward simplicity may seem completely at odds with the commonly accepted idea that maximum complexity has increased over geological time. From the broad sweep of life’s history to more specific animal clades, multiple empirical studies using quantitative assessments of configurational complexity support this trend (e.g., number of cell types, vertebral formulae, and crustacean tagmosis) (*58*, *64*, *111*, *112*). Yet, the mechanisms behind this growth remain highly debated. For instance, the zero-force evolutionary law (ZFEL) posits that, in the absence of natural selection and constraints, random mutations statistically expand the variance around the initial state, passively expanding maximum complexity over time (*113*). Downplaying the role of selection and adaptive benefits in similar way, constructive neutral evolution (CNE) theory predicts that complexity increases because neutral mutations tend to accumulate nonfunctional structures that become interdependent with other functional structures, ultimately generating highly integrated, complex, and irreversible biological systems (*114*). In contrast, other models place natural selection and adaptation at the center of the process and suggest that complexity increases through ecosystem-level complexification mechanisms that promote division of labor and specialization of parts (*91*, *115*, *116*). In the absence of consensus, it is plausible that each process operates at different scales and in concert, but, whatever the hypothesis, the growth of complexity is not incompatible with the simplicity bias because the two trends describe different aspects of evolutionary dynamics. The simplicity bias does not preclude the emergence of increasingly complex phenotypes or an increase in maximum complexity; it simply predicts that such complex forms will become progressively rare during evolution. Actually, the challenge rather lies in disentangling the respective roles of random (passive) and selective (directional) processes, which can have additive, neutral, or antagonist effects on complexity dynamics.

### Universal evolution rules?

As argued by Gould, evolution is generally understood as a process operating at the intersection of innumerable random biological and environmental factors, making it highly sensitive to historical contingencies and largely unpredictable in the long run (*117*). Within this conceptual scheme, living forms arise and evolve in multiple and erratic directions from an initially simple and irreducible configuration typical of the earliest life forms (e.g., rounded bacterial shape). Thereby, the observed growth of complexity could just result from chance such that the expansion of life into the most remote, complex, and heteromorphic areas of geometric complexity space could just be a matter of time and opportunities (*118*, *119*), rather than the outcome of specific constraints or biases. While the role of contingency cannot be dismissed, multiple examples of anatomical convergence among independent lineages yet indicate that deep physical, chemical, and biological constraints have imposed directional patterns on morphological evolution (*5*, *7*, *8*). Here, our results appear to support the prevalence of such underlying necessity at the whole biosphere scale. When combined with fossil record data, our findings suggest that life on Earth has followed the most statistically probable morphodynamical pathway, driven by the accumulation of body size variance and hierarchical complexity over geological times (*1*, *3*, *4*, *120*). In the geometric complexity space, this trend is reflected in four major phases of morphodynamical expansion and complexity increase, each associated with progressive rises in body size (Fig. 9, B and C): body elongation, densification of appendages, acquisition of mass heterogeneity, and finally, structural heterogeneity. This scalar morphodynamical sequence corresponds to the general trend recorded throughout the geological past in the following chronostratigraphic order:

1) The initial step of shape elongation associated with increasing body sizes likely occurred during the Archean, a time when life was restricted to microscopic prokaryotes that occasionally formed stromatolitic structures. Despite the challenges of fossil preservation, the bacterial fossil record from this period already reveals spherical and lenticular shapes, as well as, in some cases, filamentous organizations (*121*, *122*). Molecular clock analyses further confirm this tempo, as most bacterial phyla, including filamentous cyanobacteria and flagellate lineages, diversified during the Archean (*123*, *124*).

2) The densification of appendages likely accompanied the increase in body size identified at the end of the Paleoproterozoic (*4*, *125*), with the emergence of unicellular eukaryotes (protists) and, later, multicellular forms. Microfossils dated between 1.9 and 1.4 Ga (billion years), including 100- to 300-μm cysts with filamentous or spiny ornamentation, support this trend (*126*, *127*). This process may have begun earlier with the possible emergence of basal groups (the First Eukaryotic Common Ancestor) in the late Archean, but fossil evidence remains debated (*128*, *129*). Regardless of timing, structural densification was enabled by the evolution of cytoskeletons capable of maintaining cell architecture and anchoring pseudopods. Later, cell cohesion facilitated the rise of multicellularity and larger body sizes (*4*, *125*), further promoting structural density in the Paleoproterozoic. This is supported by branched multicellular fossils assigned to red and green algae in ~1-Ga sediments, as well as 1.63-Ga-old filamentous fossils attributed to stem groups (*130*, *131*).

3) It may be tempting to suggest that mass heterogeneity first arose in the Paleoproterozoic, alongside increases in body size (*4*) and structural densification in early protist groups. However, several lines of evidence more strongly support a Neoproterozoic origin. First, the modern organisms exhibiting the highest levels of mass heterogeneity are rhizarians (Foraminifera, Radiolaria, and Cercozoa) and centrohelids, two marine unicellular lineages whose origins are dated by time-calibrated phylogenies between the Mesoproterozoic and Neoproterozoic (*132*). Second, these protists spanning scales between 10^−5^ and 10^−4^ m share an essential innovation: the presence of rigid, fully or partly biomineralized exoskeletons that support centralized body masses and long defensive or feeding structures such as spines, axopods, or pseudopods. Although the emergence of biomineralizationin protists remains difficult to date, the earliest confirmed evidence in Rhizaria dates to ~750 million years (Cryogenian) (*133*). These observations therefore support a Neoproterozoic origin for mass heterogeneity.

4) The high structural heterogeneity observed at metric scales is largely driven by terrestrial plants (Tracheophyta) and thus necessarily arose after their terrestrialization and magnification during the Paleozoic. Adaptation to the contrasting conditions in soil and air was facilitated by a differentiation, polarization, and rigidification of aerial (branches and leaves) and hypogeous (roots) structures. As these features are most fully developed in ferns, conifers, and angiosperms that successively appeared between the Devonian and Cretaceous (*134*), we infer that the peak of structural heterogeneity was reached relatively late, likely during the Mesozoic.

At this stage, the interpretation of morphodynamical changes supports the idea that the overall geometric complexity of the biosphere has increased stepwise through time. However, disentangling the respective contribution of selective pressures (e.g., ecosystem complexification) and random processes [e.g., as proposed by the ZFEL and CNE theories (*113*, *114*)] to this trend remains highly challenging without a quantitative assessment of complexity patterns in the fossil record. With due caution, if part number, part differentiation, and part arrangement may predominate within each theoretical framework [i.e., CNE, ZFEL, and ecosystem-driven theories, respectively (*55*)], our results suggest that these mechanisms could have prevailed in distinct contexts. Beyond necessity, the relatively late emergence of organisms with high structural and mass heterogeneity also aligns with expectations from algorithmic and developmental simplicity biases: Heteromorphic forms are statistically rare, and their appearance mainly depends on a combination of fortuitous innovations (e.g., rigid biomaterials) and ecological opportunities. Likewise, the persistence of massive taxa throughout evolution, despite physical and metabolic constraints, also highlights the key role of internal innovations (*18*).

In conclusion, the morphodynamical factors exert different influences on the geometric complexity of life. Under the selective pressures imposed by particular environments and scales, functional (i.e., metabolic or other) and fabricational (i.e., physical and chemical) necessities define clear boundaries between possible and impossible living forms (*17*), guiding life’s evolution along a coherent and directional morphodynamical pathway dictated by gradual shifts (and related complexification) from “microscopic balls to branched and networked giants” (Fig. 8E). However, the body size threshold at which these constraints operate might be flexible, especially if we expect different environmental conditions on other planets or internal innovations maintaining unstable shapes at higher scales. In contrast, the computational (genetic) factor acts as a source of diversification and complexification, passively and asymmetrically shaping the likelihood of shapes achieved during evolution (*104*). By limiting the development of complex heteromorphic shapes that are statistically rare, simplicity biases render the space of geometric complexity anisotropic (Fig. 9B), with evolutionary pathways more likely in regions dominated by uniformity. When linked, these fundamental principles suggest that similar rules of complexity and directionality might be applicable to any potential life in the universe (fig. S2) (*5*, *7*).

## MATERIALS AND METHODS

### Sampling of living shapes

A panel of 944 images, featuring unicellular and multicellular organisms in solitary, aggregated, or colonial forms, was compiled from 160 sources (data S1). This collection represents 839 modern species (including duplicates due to the solitary/colonial nature of some taxa) and spans 121 prokaryotic and eukaryotic phyla reviewed in recent syntheses (*50*, *135*). On average, eight species per phylum were selected to capture the within-group variability across different (sub) classes and body sizes (fig. S1). However, sampling efforts varied between one single specimen for rarer phyla to a range of 25 to 92 species for well-represented groups like angiosperms, vertebrates, euarthropods, molluscs, cnidarians, rhodophytes, Bacillota, and Euryarchaeota. In common terms, this sampling encompasses 157 bacteria, 53 archaea, 131 unicellular eucaryotes (encompassing macroscopic syncytial organisms), 396 metazoans, 131 plants, 35 fungi, and 41 multicellular algae.

For practical reasons linked to data acquisition, our approach reduces each specimen to a silhouette equivalent to a 2D projection of the 3D shape. To preserve the maximum of spatial information and ensure a consistent analysis whatever the body plans, we used some criteria for selecting the shapes: (i) For a given taxon, the chosen orientation scheme presents, by order of importance, the longest body axis, the maximum number of structures, and then the maximum surface area; (ii) the orientation scheme remains the same within a same phylum or class; (iii) for motile taxa, the limbs or appendages are unfolded and not overlapping, favoring symmetrical representations avoiding potential bias in body posture (see the Postural test section). However, exceptions have been made for most tree and fungi taxa, as branches, roots, or mycelium inevitably overlap in 2D projections. Moreover, their gigantic size, the unavailability of images showing the hypogeous parts, and the ontogenetic variability of shapes complicate sampling. For fungi, we therefore focused on specific parts summarizing different growth stages and structures (e.g., mycelium, cord system, and sporophores) rather than whole shapes. For most trees, we assembled images of aerial parts and roots systems reconstructed manually from various sources, by ensuring accuracy in the root typology of species.

### Body size and ecological data

Several data have been gathered for each studied specimen (data S1 and fig. S1). First, their body size has been reported as the longest body length of the shape, avoiding complex biovolume calculations due to limited 3D data for most specimens. Body lengths were obtained from direct measurements or literature data and then categorized into 10 orders of magnitude, ranging from 10^−7^ to 10^2^ m. The specimens have been classified into three levels of hierarchical complexity (*1*): prokaryotes, unicellular eukaryotes, and multicellular eukaryotes. Each level was also subdivided into solitary or aggregated/colonial shapes. Last, the specimens have been classified into four ecological groups: (TER) Terrestrial taxa inhabiting gaseous environments, (AQU) Aquatic taxa living in liquids like freshwater or seawater, (ORG) Epi- or endobiontic taxa, often symbiotic or parasitic, living in organic fluids or tissues, (HYP) hypogeous and endobenthic taxa inhabiting soil or marine sediment. Those occupying two environments (e.g., aerial branches and hypogeous roots of plants) were considered as 50–50%.

### Image processing

The images of selected taxa have been converted into vectorized black silhouettes on Adobe Illustrator CS6, with a dynamic vectorization procedure to remove the background elements. When necessary, the contours were smoothed and the incomplete or intersecting parts were redesigned manually for clarity. To emphasize structural information over textural details, the resolution of the finest features was limited to ~1‰ of the total body length (i.e., one pixel thick). The resulting silhouettes were standardized to a body length of 860 pixels, centered in a 1000 pixel–by–1000 pixel frame, and saved as bitmap files (Fig. 2). The topological skeleton of each silhouette was extracted using the “skeletonize” function of FIJI (*136*), without an additional skeletal pruning algorithm. This method iteratively erodes the shape’s contours by removing pixels until an equidistant and median linear structure remains.

### Biomorph generation procedure

Exploring the spectrum of possible forms for a given body plan generally requires a mathematical model based on generative parameters linked to biological growth processes, or at least a combinatorial framework of morphological descriptors whose regular variation produces a finite or infinite set of possible forms (simulated or not) (*20*, *22*, *25*, *54*, *86*). In practice, these parameters represent the axes of an *n*-dimensional theoretical space in which the position of extent taxa can be graphically visualized and compared to the field of possible shapes. Here, we addressed this theoretical issue unconventionally since the fractal descriptors obtained through the box-counting method are statistical indices of shape complexity that are not directly applicable to shape generation (*44*). Furthermore, developing a general mathematical formulation of the space of possibilities remains challenging, as the relationship between fractal dimension and lacunarity is nonmonotonic and depends on the specific characteristics of studied objects (*46*, *68*–*70*). To overcome this limitation, we heuristically explored the spectrum of possible combinations of fractal parameters by generating and measuring 15,391 images of unextant “biomorphs” (Fig. 3 and data S2). As for the living shapes, the biomorphs were standardized to a maximum length of 860 pixels and centered in a 1000 pixel–by–1000 pixel frame. Note that neural network–based generative models such as GANs (*137*), VAEs (*138*), or more recent DDPMs (*139*) and CreativeGAN (*140*) could be used to generate synthetic biomorphs. We however preferred using more conventional methods to keep an explicit control on the properties of the generated biomorphs. Validating the plausibility and the variability of the generated shapes using neural networks would also be another research project in itself.

First, 1904 biomorphs have been modeled using the Gielis’ superformula (*51*) implemented on the Superformula Plotter software (*141*). This model generates shapes that mimic biological objects and combine both rotational symmetries and asymmetrical components. Derived from a superellipse equation, it uses a polar coordinate system in which a circle of radius *r* is deformed as a function of a rotation angle ϕ according to six parameters

$$r\left(\phi \right)=f\left(\phi \right){\left[{\left|\frac{1}{a}\text{cos}\left(\phi \frac{m}{4}\right)\right|}^{n2}+{\left|\frac{1}{b}\text{sin}\left(\phi \frac{m}{4}\right)\right|}^{n3}\right]}^{-\frac{1}{n1}}$$

*m* defines the order of rotational symmetry. *n_2_* and *n_3_* influence the homogeneity (*n_2_* = *n_3_*) or heterogeneity (*n_2_* ≠ *n_3_*) of shape corners defined around the rotational axis, as well as the concavity (*n_2_* = *n_3_* < 2) or convexity (*n_2_* = *n_3_* > 2) of edges. *n_1_* affects the sharpness or smoothness of corners, while *a* and *b* accentuate the magnitude of previous deformations. Further shape modifications are produced by varying a facultative parameter *p* smoothing the shape contours or by adding a function *f*(ϕ)^33^. For *f*(ϕ) = 1, the resulting shape remains unchanged, but for other terms such as ϕ*, sin*(ϕ)*, cos*(ϕ), (ϕ)*^1/2^*, *sin*(ϕ)*^2^*, *cos*(ϕ)*^2^*, 1 *+ sin*(ϕ)*^2^*, *1 + cos*(ϕ)*^2^*, *atan*(ϕ), bilateral symmetries, asymmetries, or spiral curves can be generated.

In the second step, 9959 biomorphs were manually generated using vector drawing procedures on Adobe Illustrator CS6 (Fig. 3A). This method completes the previous one by producing bacterial forms with radial (peritrichous) or polarized (lophotrichous) organizations and very thin appendages that are difficult to produce with the Gielis Superformula. These shapes are based on the combination of an elliptic body mass to appendages systems. The shape of the body mass is defined by the following: a rotundity index (*R*_*M*_), calculated as a height-to-width ratio ranging from 0.1 for very compressed ellipses to 100% for rounded shapes; its relative size (*S*_*M*_), ranging from 0 for a lacking mass to 100% if it covers the whole shape length; its parallel or perpendicular orientation (*O*_*M*_) relative to the major body axis. The body mass is then complemented by a primary appendage system (*PAS*) comprising one to several straight lines. Each line is 430 px long and follows a radial and equiangular organization around an anchor point (*A*_*P*_) located at the center or periphery of the mass. The configuration of the *PAS* is modulated by: the number of appendages (*N*_*P*_), ranging from 1 to 2000; the rotational distribution of appendages (*R*_*oP*_), varying from 2.5° to 360°; the thickness of appendages (*T*_*P*_), varying from 0.1 to 100% of the shape length. *PAS* was optionally modified according to a deformation criterion *D*_*P*_, either by stretching its length up to reach 860 px in length and/or by compressing its width down to the body mass height or inferior values. To finish, a secondary appendages system has been intercalated between the primary structures. This second system is defined by the number of secondary appendages (*N*_*S*_) (from 0 to 1500), their rotational distribution (*R*_*oP*_) (from 0.25° to 360°), and its relative size (*S_*S*_*) (from 1.5 to 100% of the shape).

Whatever the model used, we varied the generative parameters*,* initially with regular increments across their variability ranges, followed by random sampling. On the basis of continuous feedback from fractal measurements of the generated biomorphs, we then fine-tuned the parameters to achieve exotic shapes, ultimately filling the empty areas or pushing back the limits of the geometric complexity space. To get additional silhouettes that are more challenging to produce mathematically, 1138 and 2390 biomorphs, respectively, generated with the Gielis’ model and the vector method were further transformed using FIJI and Adobe Illustrator CS6: (i) by adding a central mass to the spirals; (ii) by skeletonizing the silhouettes with FIJI to obtain threadlike shapes; and (iii) by thickening the resulting skeletons. The total number of simulations was capped at 15,391 biomorphs because the space structure remained stable even with the addition of further shapes.

### Fractal analysis and calibration

The fractal dimension *D*_*F*_ of geometric objects expresses how their measures *N*ε (e.g., perimeter, area, and number of subparts) change with observation scale (ε). This scaling rule relies on the fact that finer details emerge at smaller scales, leading to higher measure values as resolution increases (*45*). For self-regular shapes, this relationship follows a power law (*44*): $N\varepsilon \propto \varepsilon^{D_{F}}$ such as $D_{F}=\frac{\text{ln}N\varepsilon }{\text{ln}\varepsilon }$. For natural shapes lacking self-similar pattern, a statistical approximation of *D*_*F*_, termed *D*_*B*_ (or Minkowski-Bouligand dimension), can be determined using box-counting methods (*44*, *48*, *49*, *142*). The method involves overlaying successively smaller grids on the image and counting the number of black boxes containing the shape to estimate *N*ε at each grid resolution ε (with box size = 1/ε) (Fig. 2). *D*_*B*_ is then calculated as the slope value of the linear regression of ln *N*ε against ln ε in a bivariate plot (*44*). More formally, $D_{B}=\underset{\varepsilon \to 0}{\text{lim}}\left(\frac{\text{ln}N\varepsilon }{\text{ln}\varepsilon }\right)$. In addition, we calculated the lacunarity parameter *L* to quantify the heterogeneity of shapes (*46*). During the box-counting procedure, lacunarity at a given grid resolution ε (noted λε) is determined as $\lambda \varepsilon ={\left(\frac{\sigma }{\mu }\right)}^{2}$, where σ is the SD and μ is the mean number of black pixels per box at resolution ε. The successive λε values are averaged to obtain the overall lacunarity of the shape denoted *L* (Fig. 2).

We measured *D*_*B*_ and *L* of silhouettes and topological skeletons using the box-counting method implemented in the Fraclac plugin of FIJI (*143*) (data S1). A standard scaling method based on a linear progression of box size was applied. To avoid noise and inconsistent measures at extremely high or low grid resolutions, we limited the range of box sizes from 4 to 200 pixels (i.e., 0.4 to 20% of the image size) (*142*). For each silhouette and corresponding skeleton, we performed 120 analyses of *D*_*B*_ and *L* by applying 10 image rotations of 36° and 12 initial grid positions. The averages provide therefore four parameters *D*_*SI*_, *L*_*SI*_, *D*_*SK*_, and *L*_*SK*_ for each shape. The mean SDs associated with the four parameter measurements are 0.03, 0.02, 0.04, and 0.04, respectively.

We validated the robustness of results by comparing the measured *D_B_* values to theoretical *D*_*F*_ values of 24 self-similar forms (Fig. 1B). Despite a strong correlation between *D*_*B*_ and *D*_*F*_ (Spearman *rs* = 0.98; *P* < 0.001), *D*_*B*_ values for dense shapes close to uniform surfaces were systematically lower by −0.1 than *D*_*F*_. Hence, we corrected the *D*_*B*_ values using a power law model (*R*^2^ = 0.99) such as $D_{B\left(\text{corr}\right)}=0.4565\times {D_{B}}^{1.8027}+0.55436$. This calibration better scales *D*_*B*_ values between 1 and 2, improving discrimination of denser shapes. In the text and figures, all *D*_*SI*_ and *D*_*SK*_ values are corrected values.

### Postural tests

Before analyzing the extant living forms, we assessed the influence of posture on the calculation of fractal parameters in articulated (often mobile) taxa. Thirty-six human silhouettes in different postures have been generated on Adobe Illustrator CS6, featuring different degrees of limb bending and crossing, both symmetrically and asymmetrically (fig. S2). Each silhouette was standardized to a body length of 860 pixels and centered within a 1000 pixel–per–1000 pixel frame. These silhouettes were then analyzed using the same protocol as detailed above. The results indicate that the skeleton-based metrics (*D*_*SK*_ and *L*_*SK*_) remain relatively consistent across different body postures, with 2σ variations not exceeding ±0.03. In contrast, silhouette-based metrics (*D*_*SI*_ and *L*_*SI*_) are more sensitive to postural changes (2σ = 0.1), as limb bending and overlap can artificially inflate the apparent mass, leading to higher *D*_*SI*_ and lower *L*_*SI*_ values. Conversely, staggered or asymmetrical postures tend to decrease *D*_*SI*_ and increase *L*_*SI*_. For consistency, we therefore preferentially selected specimens with symmetrical, natural postures and fully extended, nonoverlapping appendages.

### Space visualization

The 3D scatter plots of *D*_*SI*_, *D*_*SK*_, *L*_*SI*_, and *L*_*SK*_ displayed in Fig. 4 (A to C) have been performed with Plotly Chart Studio. Note that two disk shapes have been excluded from the scatter plots and the rest of the analyses because their topological skeleton is a point (*D*_*SI*_ = 2; *D*_*SK*_ = 0), which classifies them as singularities outside the space of parameters.

To visualize the distribution of data points in a bidimensional projection, we applied an Isomap (*144*) ordination method to the standardized parameters using the Orange v.3.38 software. Unlike classical principal components analysis (PCA), which relies on assumptions of linearity, normality, and homoscedasticity, this nonlinear dimensionality reduction technique captures the structure of the underlying manifold (e.g., the curvature shown in Fig. 4A) and better considers neighborhood information (*145*), which is particularly relevant in our case. In comparison to other manifold-learning methods such as LLE or UMAP, it better preserves the global structure of datasets (*145*), ensuring a relevant appraisal of shape transitions and limits in the geometric complexity space (the end-members are identified from 3D scatterplots). The Isomap algorithm first computes a neighborhood graph in which each data point is connected to its *k* nearest neighbors (here, *k* = 100). The geodesic distance between each pair of data points is then estimated by finding the shortest path distances in the graph. Last, a principal coordinate analysis is applied to the matrix of geodesic distances. For comparison, we also performed a PCA on the correlation matrix, which gives similar results (fig. S3 and table S3). To visualize the distribution of data points within the resulting space (Figs. 5 to 7), Kernel density maps applying a Gaussian function with a radius of 0.15 per point have been performed on PAST v.4.11 (*146*).

### Disparity and statistical analysis

Owing to the non-Euclidean properties and the curved manifold of the parameter space, we estimated the complexity disparity of groups by computing the ratios of generalized variances (*66*) on RStudio v.4.4.2 (Figs. 6B and 8D and table S1). The generalized variance of each group is defined as the determinant of the covariance matrix, and to ensure homogeneity, the ratios are systematically expressed by fixing the group with the highest generalized variance value as the common denominator. The ratios were calculated with a rarefaction procedure (*n* = 50 samples) and 1000 bootstraps to minimize unequal sampling effects. The results are reported with boxplots performed on RStudio v.4.4.2 using the package ggplot2 (v.3.5.0).

Before any statistical test performed on RStudio v.4.4.2 (Table 1 and tables S1 and S2), the normality and multinormality of data of each subgroup were checked using the fBasics and mvnormtest functions. Given the nonnormality of the data, we systematically applied nonparametric tests. For univariate analyses, we compared the groups medians (Wilcoxon, Kruskal-Wallis, and Dunn tests) and variances (Levene’s test) with the Matrix, car, and dunn.test packages. For multivariate analyses, we assessed group differences by comparing medians (multivariate Wilcoxon test), centroids (PERMANOVA), covariance matrices (Box’s M test), and group similarity (ANOSIM) with the coin, biotools, and rcompanion packages and the function adonis2 of vegan.

### Artificial intelligence assistance

We used ChatGPT versions 4 and 5 to refine the language and grammar in some parts of the text. The prompt was “Can you improve the English style of this scientific text while maintaining the meaning?” All results were carefully reviewed (selected or not) for the final text version.
