# Supplementary material for: Mapping life’s disparity and evolutionary constraints in a geometric complexity space
Source: Sci Adv. 2026 Jan 7;12(2):eaea6945. doi: 10.1126/sciadv.aea6945 (PMC12778060; doi:10.1126/sciadv.aea6945)
Supplement: Supplementary file 1 — Figs. S1 to S3 Legends for tables S1 to S3 Legends for data S1 and S2 [file sciadv.aea6945_sm.pdf]

Science Advances
AAAS

Supplementary Materials for

Mapping life's disparity and evolutionary constraints in a geometric complexity space

Guillaume Dera *et al.*

Corresponding author: Guillaume Dera, guillaume.dera@utoulouse.fr.

Sci. Adv. **12**, eaea6945 (2026)
DOI: 10.1126/sciadv.aea6945

The PDF file includes:

Figs. S1 to S3
Legends for tables S1 to S3
Legends for data S1 and S2

Other Supplementary Material for this manuscript includes the following:

Tables S1 to S3
Data S1 and S2

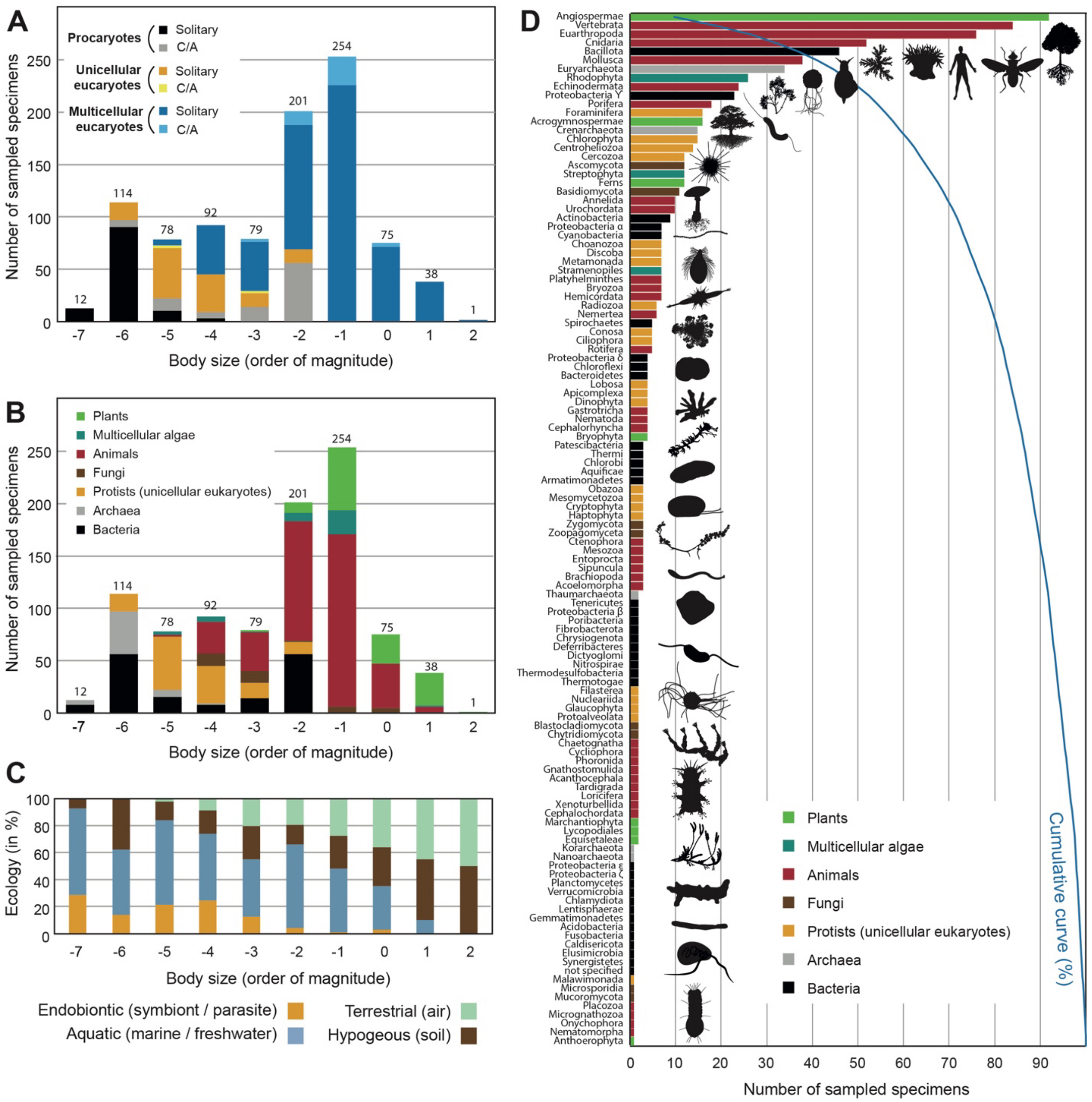

Fig. S1. Sampling of modern living forms. For each body size level (expressed in order of magnitude of the longest body axis), the 944 living shapes are classified as a function of hierarchical complexity (**A**), general biological groups (**B**), and environment (**C**). (**D**) Ranking of phyla according to the number of analyzed taxa. C/A for colonial or aggregated shapes.

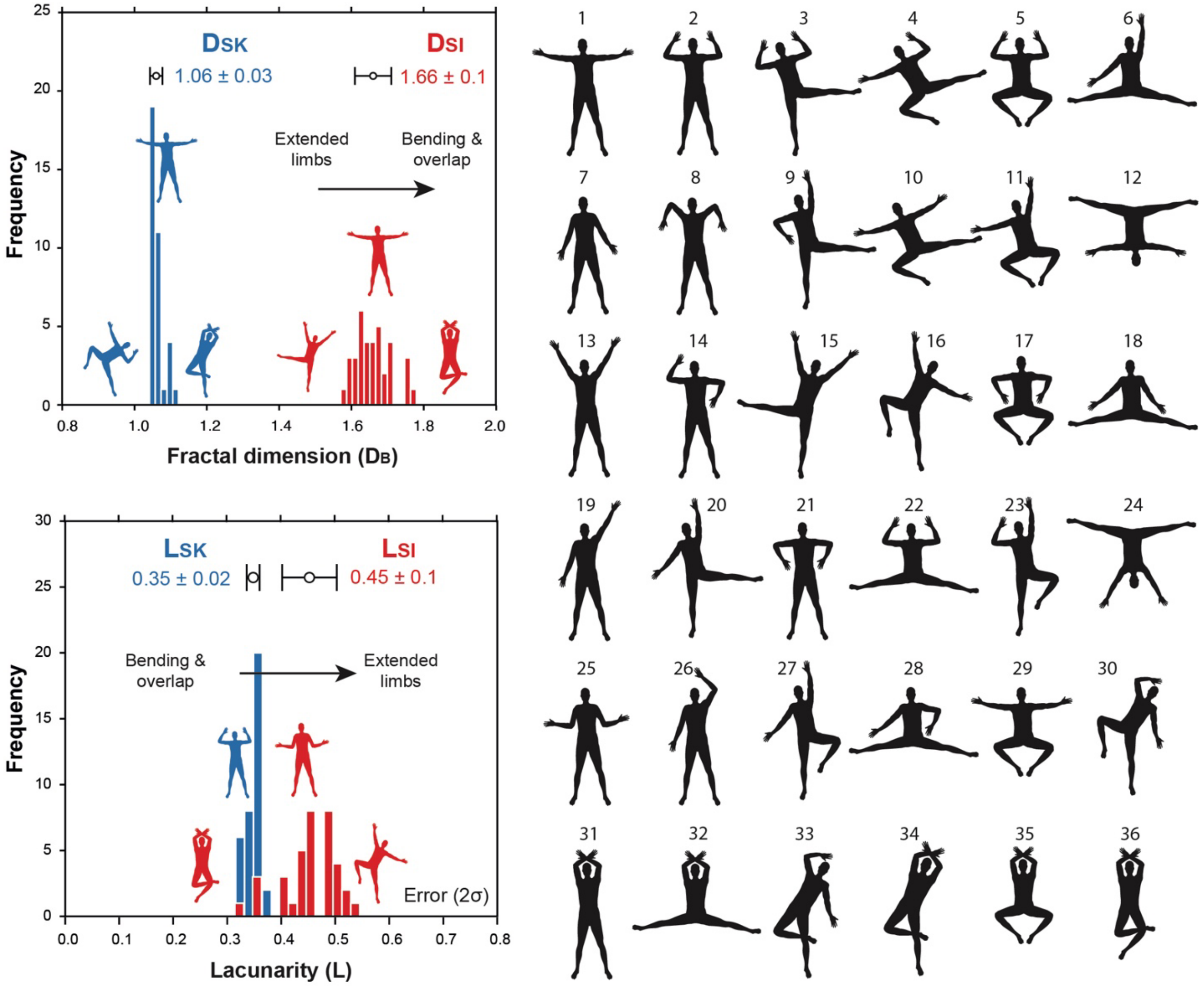

Fig. S2. Influence of body posture on the calculation of fractal parameters.

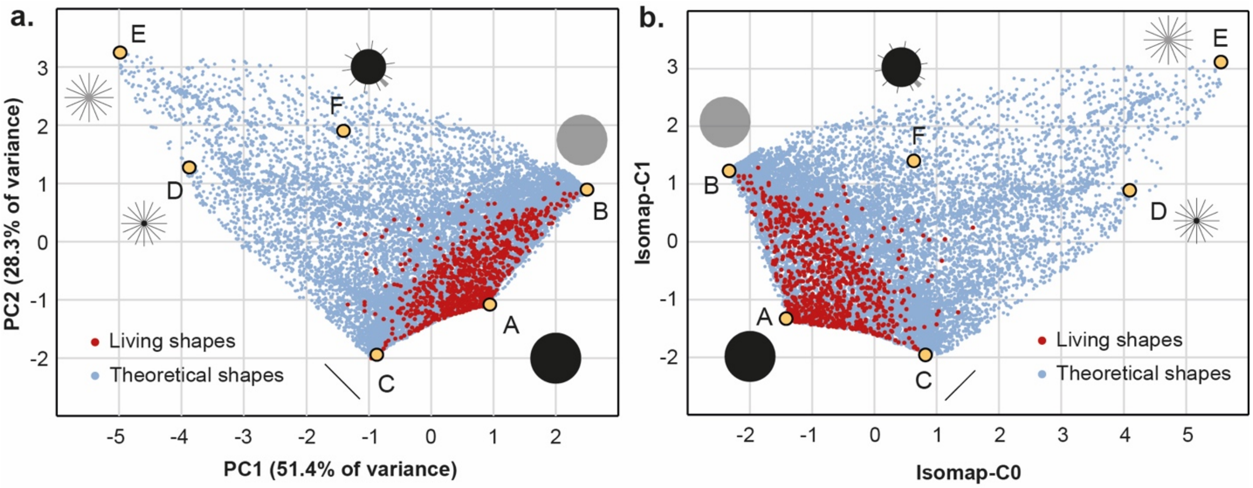

Fig. S3. PCA and Isomap ordination methods applied to the total dataset (including biomorphs and living forms). (**A**) PCA based on the correlation matrix of fractal parameters. (**B**) Isomap of standardized fractal parameters values.

Table S1. Multivariate analyses of fractal parameters.

Table S2. Univariate analyses of fractal parameters.

Table S3. Isomap and PCA ordination results.

Data S1. Dataset of living forms.

Data S2. Dataset of theoretical biomorphs.
